# Effects of Portland Cement and Polymer Powder on the Properties of Cement-Bound Road Base Mixtures

**DOI:** 10.3390/ma13194253

**Published:** 2020-09-24

**Authors:** Przemysław Buczyński, Marek Iwański, Grzegorz Mazurek, Jakub Krasowski, Maciej Krasowski

**Affiliations:** Department of Transportation Engineering, Faculty of Civil Engineering and Architecture, Kielce University of Technology, Al. Tysiąclecia Państwa Polskiego 7, 25-314 Kielce, Poland; iwanski@tu.kielce.pl (M.I.); gmazurek@tu.kielce.pl (G.M.); jkrasowski@tu.kielce.pl (J.K.); krasowski4.mk@gmail.com (M.K.)

**Keywords:** road base layer, bound mixture, Portland cement, polymer powder, design of experiment, fracture toughness, hydraulic binder, stiffness modulus

## Abstract

This article presents the test results for the physical and mechanical properties and fracture toughness of polymer-modified hydraulically-bound mixtures (HBM) produced with Portland cement for road base layers. The modifier used was a redispersible polymer powder (RPP) based on a vinyl ethylene acetate (EVA) copolymer obtained by spray drying. A three-level full factorial design with two factors was applied to determine the contents of Portland cement and polymer powder in the cement-bound mixture (CBM). Both Portland cement and polymer powder were added at three levels: 0%, 2%, and 4%. The assessment included basic physical properties (water absorption, density, and bulk density) and mechanical properties (stiffness modulus, axial compressive strength, and indirect tensile strength) of the CBM. Particular attention was paid to the assessment of fracture toughness in the semi-circular bending test. The results of the research show that polymer powder positively influenced the mechanical properties of CBM by improving its cohesion while maintaining its stiffness. Another benefit coming from the use of polymer powder was the CBM’s increased resistance to cracking, which is the desired characteristic from the perspective of pavement durability.

## 1. Introduction

The base layer is responsible for structural support and load distribution in the pavement system. Soil conditions and anticipated traffic loads determine which materials are used in paving construction. According to Polish guidelines [1], flexible or semi-rigid bases can be made of well graded aggregates, asphalt concrete, emulsion or foam cold recycled mixtures, or bound mixtures. Rigid base courses are made of the cement-bound materials defined as mixtures of a granular material or sand and cement, compacted at optimum moisture content. Applied primarily in paving construction, they are also successfully utilized as a mass foundation material [2].

Expanding numbers of vehicles are creating a necessity to enhance pavement durability [3]. The base layer is subjected to forces that generate tensile stresses under the wheel load. If the permissible number of standard axes is exceeded, fatigue failure causes bottom-up cracking. Over time, flexible and semi-rigid bases experience permanent deformation, while rigid bases crack. This form of distress is transferred through the entire pavement structure up to the wearing course [4,5]. To address the increasing requirements for road durability, quality parameters are being improved and new alternative solutions are being sought [6,7,8].

Various material solutions have been used to provide the base course with improved mechanical parameters, for example, by modifying bitumen properties with polymers—plastomers or elastomers. Plastomers have properties that cause bitumen to harden and improve high-temperature characteristics [9]. Elastomers increase resistance to low-temperature cracking, while maintaining the resistance to high-temperature deformation [10]. Various additives are used to improve bitumen foaming characteristics [11,12]. Fine-grained minerals are added to cold recycled mixtures [13,14], whereas, in low-temperature mixtures, mineral fibers are used [15].

In the case of rigid bases, efforts are made to adapt the solutions used in general construction, where increased useful loads force designers and contractors to improve the durability of concrete structures while reducing the dead weight of elements produced. One of the methods applied is the modification of concrete with polymers to obtain a polymer–cement concrete (PCC). The benefits of using polymers include increased tensile strength and better workability of the mixture. Positive results of studies on polymers in mortars and concrete mixtures are reported in [16,17].

Cement-bound mixtures (CBM) can be used to increase the pavement load-bearing capacity. The stiffness modulus in CBM is higher than in traditional bases made of aggregates, asphalt concrete (AC), and high-modulus asphalt concrete (HMAC) [18]. On the other hand, the high stiffness modulus of bound mixtures causes a high risk of shrinkage cracking and crack propagation through the bituminous mixture layers, manifested as reflective cracks. Researchers dealing with hydraulically-bound mixtures and recycled cold mixtures with Portland cement are testing various additives or components that may reduce stiffness while maintaining the required service life. Table 1 summarizes additives and binders used in the road base bound mixtures.

In addition to Portland cement, the other most commonly used binders include bitumen emulsion and foamed bitumen, applied at 1.5–5.5% of the bitumen extracted from the binder. Bituminous binders reduce the stiffness of the mixture while maintaining proper cohesion and increased deformability. However, preliminary research results [26] show that the polymer powder used in cement- and foamed bitumen-bound mixtures provides high cohesion without increasing the stiffness.

This study analyzed the physical and mechanical properties of CBM in terms of the polymer and cement contents. A typical redispersible polymer powder—ethylene-vinyl acetate (EVA)—was used in the study. The copolymer of ethylene and vinyl acetate was selected based on the authors’ preliminary findings concerning the effects of various polymers on the properties of cold recycled mixtures with foamed bitumen [26]. The use of EVA copolymer allowed achieving high cohesion at stiffness maintained at the same level [26]. Used in the alkaline environment of cement pastes, EVA was found to minimize the detrimental saponification reaction [27]. Unlike polyvinyl alcohol (PVA) [27,28], EVA is considered to have no adverse effects on cement concrete. Particular attention was paid to crack resistance. Excessive quantity of cement gives a quick effect of increasing load-bearing capacity but causes stiffening of the layer and potential for cracks. The use of polymers in CBM results in the system that combines the properties of both the polymer and the CBM. This combination results in increased cohesion, flexibility, and ductility of the mixture and reduced risk of shrinkage crack formation. The results of this study add to the existing scope of knowledge about cement-bound mixtures and their application as the base layer in paving construction.

## 2. Materials

### 2.1. Polymer Powder

**Figure 1:**
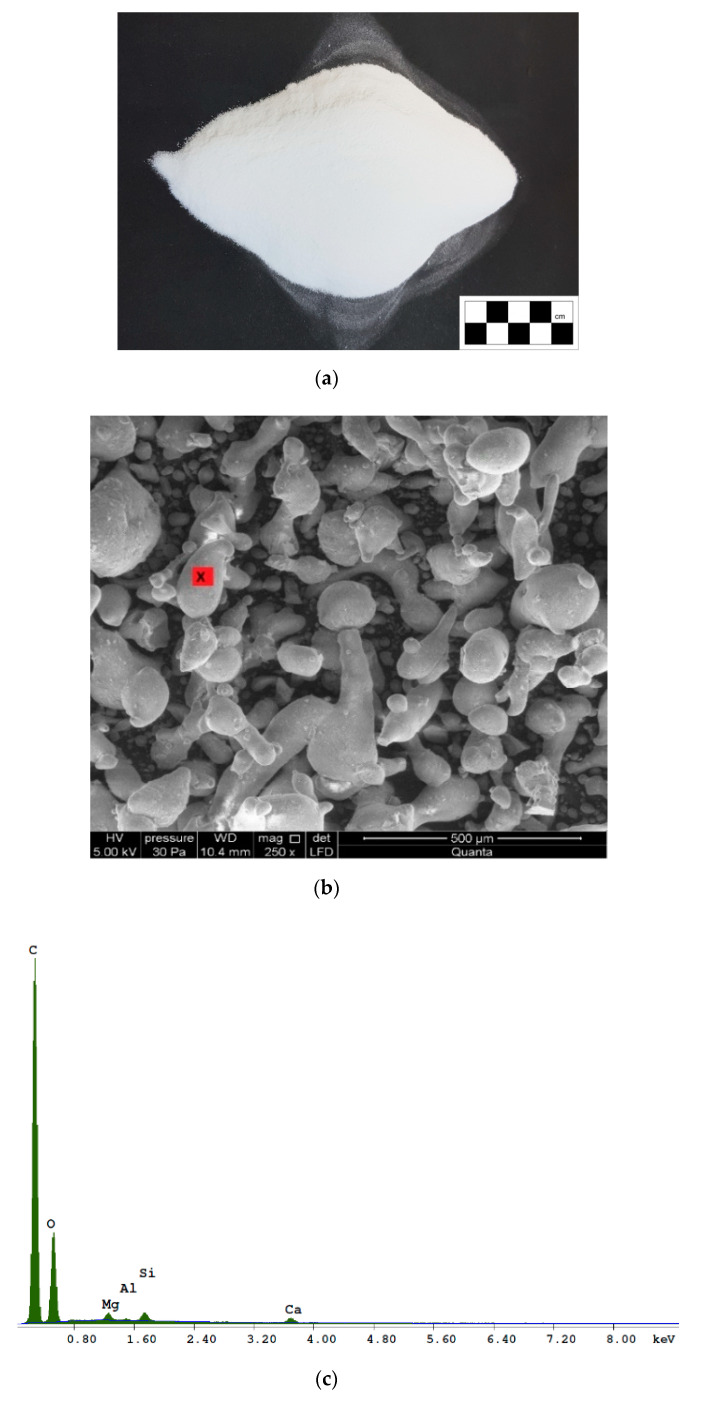
Polymer powder: (**a**) redispersible polymer powder EVA; (**b**) microstructure; and (**c**) chemical composition. The red marker indicates the point at which chemical analysis was conducted.

The redispersible polymer powder used in this study was a thermoplastic copolymer [27], ethylene vinyl acetate (EVA) [29], produced as a white powder (Figure 1a) from polymer emulsion [30] by employing a series of processes including heat treatment and spray drying [30,31]. The components used in EVA polymerization [32] are listed in Table 2.

The main advantage of using polymers in CBM is the improvement of the mechanical properties of the mixture. This effect is achieved with a network of bonded particles forming a continuous polymer phase [27].

The microstructure and chemical composition of the redispersible polymer powder were determined using a scanning electron microscopy and X-ray dispersion spectrometry SEM/EDS. The material was observed in low vacuum (pressure: 30Pa). The composition of the redispersible polymer powder was determined using the X-ray microanalyzer (EDS). Examples of the powder microstructure are shown in Figure 1, whereas the chemical composition of the polymer powder is shown in Table 3. Detailed information on the polymer powder used in this study can be found elsewhere [26].

### 2.2. Aggregate

Aggregates selected for this study were the standard mineral materials used in the production of CBM mixtures for base courses [33], derived from dolomite rocks in the Świętokrzyskie Region. Two different particle sizes were used. The aggregate with a particles size of 0/31.5 mm was marked as (J). Table 4 shows the basic parameters of the material.

The well graded dolomite aggregate with a particle size of 0/4 mm was marked as (L). The basic geometrical and physical properties of the 0/4 mm aggregate are shown in Table 5.

### 2.3. Hydraulic Binder

The CBM mixture was manufactured with class I Portland cement characterized by compressive strength of 42.5 with high early strength “R” as per EN 197-1 [42]. The binder was marked as (C). The basic properties of the cement are compiled in Table 6.

## 3. Experimental Design

To find a mathematical model for investigating the relationships between the output variable “*y*” and the input variables “*x_i_*” using a general formula, a three-level full factorial experiment design with two factors was used. The most adequate function of the response variable was identified based on a literature review [46]. The following mathematical model was selected:

(1) $$y=b_{0}+\sum_{i=1}^{n}b_{i}\cdot x_{i}+\sum_{i=j=1}^{n}b_{i=j}\cdot x_{i}\cdot x_{j}+\sum_{i=1}^{n}b_{ii}\cdot x_{i}^{2}$$

The response surface of the selected model was a second-order polynomial, which can be written as an equation with two factors (*x*_1_, *x*_2_) [47]:

(2) $$y=b_{0}+b_{1}\cdot x_{1}+b_{2}\cdot x_{2}+b_{3}\cdot x_{2}\cdot x_{1}+b_{4}\cdot x_{1}^{2}+b_{5}\cdot x_{2}^{2}$$

where *x*_1_ is Portland cement (CEM) content in the CBM mixture (%); *x*_2_ is polymer powder (RPP) content in the CBM mixture (%); and *b*_0_*–b*_5_ is experimental coefficients.

**Figure 2:**
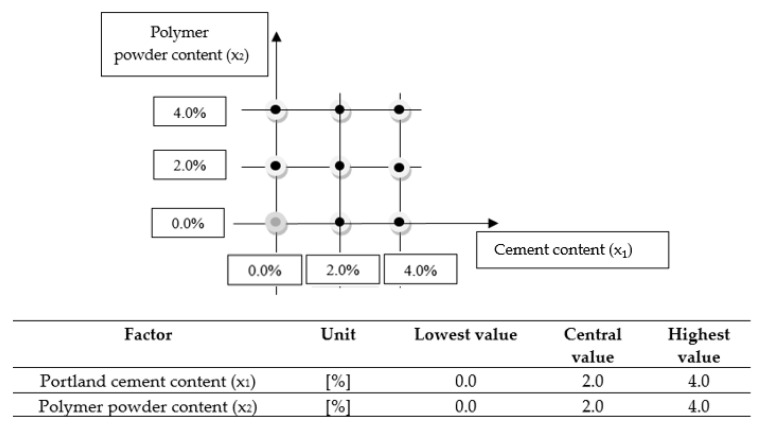
Design of experiment.

Figure 2 shows the design of the experiment.

Eight different mixtures (CBM) were obtained under the adopted experimental design. The mixtures were coded for analysis by the number of controlled factors. The mixtures are described in Table 7.

The maximum amounts (4%) of cement and polymer used in the experiment comply with the requirements for the maximum quantity of the binder in cement-bound mixtures [33]. In addition, the cement content of 4% falls within the limits of binder amounts used in similar base layer mixtures [5,19,20,21,22,23,24,25]. The quantity of the polymer powder corresponds to the cement content adopted in the design. Particular combinations, for example 4% cement and 4% polymer, result from the adopted three-level factorial design with two factors (Figure 2).

The applied experimental design allowed presenting the results in the form of regression models. The models investigated the relationships between the CEM content and RPP content factors and the properties under analysis. The extreme combinations of the CEM and RPP contents showed the changes in the properties investigated and the influence of the interaction between the factors. Mixture C0P0 was not used in the analysis.

Regression equations (response surfaces) were used to determine the optimum contents of CEM and RPP for maximum resistance to cracking at minimum or maximum stiffness.

## 4. Testing Plan

First, the cement-bound mixture (CBM) modified with polymer powder was tested for physical and mechanical properties, i.e., bulk density (saturated surface dry (SSD)) of the specimen (*ρ_bssd_*) [48], water absorption (*n_w_*) [49], stiffness modulus (*S_m_*) in the IT-CY test [50], 28-day compressive strength (UCS) [51], and indirect tensile strength (ITS) [52].

In the following step, a test was carried out as per EN 12697-44 [53] to determine fracture toughness (*K_IC_*) of the CBM.

### 4.1. Physical and Mechanical Properties

#### 4.1.1. Bulk Density—SSD

Bulk density is determined as mass per unit volume, including air voids, of a specimen at known test temperature [48]. Bulk density (SSD) of the specimen (*ρ_bssd_*) should be calculated to the nearest 0.001 Mg/m^3^ as follows:

(3) $$\rho_{bssd}=\frac{m_{1}}{m_{3}-m_{2}}\times \rho_{w}$$

where $\rho_{bssd}$ is bulk density (SSD) in megagram per cubic meter (Mg/m^3^); *m*_1_ is the mass of the dry specimen in grams (g); *m*_2_ is the mass of the specimen in water, expressed in grams (g); *m*_3_ is the mass of the saturated dry surface of the specimen, expressed in grams (g); and *ρ_w_* is the density of water at the test temperature in megagram per cubic meter (Mg/m^3^).

#### 4.1.2. Water Absorption by Weight (*n_w_*)

Water absorption [49] is the mass and volume of water absorbed by a specimen when immersed in water for 24 h at +25 ± 5 °C and then dried to constant mass. Water absorption (*n_w_*) is calculated as a percentage (m/m) to the nearest 0.1% as follows:

(4) $$n_{w}=\frac{m_{1}-m}{m}\cdot 100$$

where *n_w_* is the water absorption (%), *m*_1_ is the mass of the water-saturated specimen (g), and *m* is the mass of the dry specimen (g).

#### 4.1.3. Stiffness Modulus (*S_m_*)

**Figure 3:**
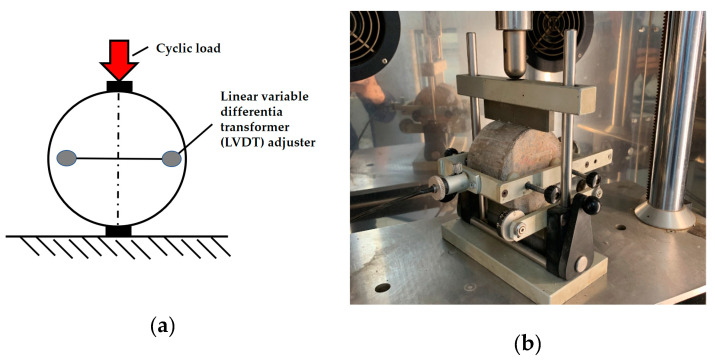
An example of a force applied to the specimen and the loading scheme: (**a**) testing scheme (source: P. Buczyński); and (**b**) set-up for fracture toughness test (laboratory at the Kielce University of Technology, Faculty of Civil Engineering and Architecture) (source: M. Krasowski).

The stiffness modulus was determined to the requirements of the EN 12697-26 standard [50], Annex C, by measuring vertical and horizontal displacements at mid-height of the specimen under controlled application of the load. The horizontal displacement was 5 ± 2 µm, and the diameter of the test specimens was 101.6 mm. The load increment was 124 ± 4 ms. The loading scheme and an example of a force pulse are shown in Figure 3.

The stiffness modulus can be calculated from formula, and Poisson’s ratio from Equation:

(5) $$Sm=\frac{F\cdot (\nu +0.27)}{z\cdot h}$$

(6) $$\nu =3.59\cdot \frac{z}{\Delta V}-0.27$$

where *S_m_* is stiffness modulus of the specimen (MPa); *F* is maximum force applied to the specimen (N); *ν* is temperature-dependent Poisson’s ratio; z is amplitude of the horizontal displacement of the specimen under loading (mm); *h* is specimen thickness (mm); Δ*V* is maximum vertical displacement of the specimen (corresponding to the maximum horizontal displacement) (mm).

Analysis of Formula (5) indicates that the increase in the transverse deformation and the thickness of the specimen reduces internal stresses in the pavement and hence the stiffness modulus *S_m_*. The stiffness modulus *S_m_* was tested at temperatures of 10 and 25 °C after the curing period of 28 days.

#### 4.1.4. Compressive Strength (*R_C_*)

Axial compressive strength (*R_C_)* was determined on cylindrical specimens prepared with the Proctor method following the requirements of EN 13286-50 [54]. The test temperature was 25 ± 3 °C as per PN-EN 13286-41 [55]. The 28-day compressive strength was determined from formula:

(7) $$R_{C}=\frac{F}{A_{C}}$$

where *R_C_* is the compressive strength of cement bound specimens (MPa), *F* is the maximum transferred force (N), and *A_C_* is the cross-sectional area of the cement bound specimen (mm^2^).

#### 4.1.5. Indirect Tensile Strength (*ITS_DRY_*)

The *ITS_DRY_* test [52] of the cement-bound mixture was performed on Marshall specimens with a diameter of 101.6 ± 0.3 mm and a height of 62.5 ± 2.5 mm cured for 28 days at a relative humidity of 40–70% and test temperature of +25 °C. The tensile characteristics were evaluated by placing the specimen between two loading strips and applying a compressive load at a constant deformation rate of 50 ± 2 mm/min. The indirect tensile strength ITS is calculated from formula:

(8) $$ITS_{DRY}=\frac{2\cdot P}{\pi \cdot h\cdot D}$$

where *P* is the maximum load at failure, *h* is the specimen height (mm), and *D* is the specimen diameter (mm).

### 4.2. Crack Propagation in the SCB Test

**Figure 4:**
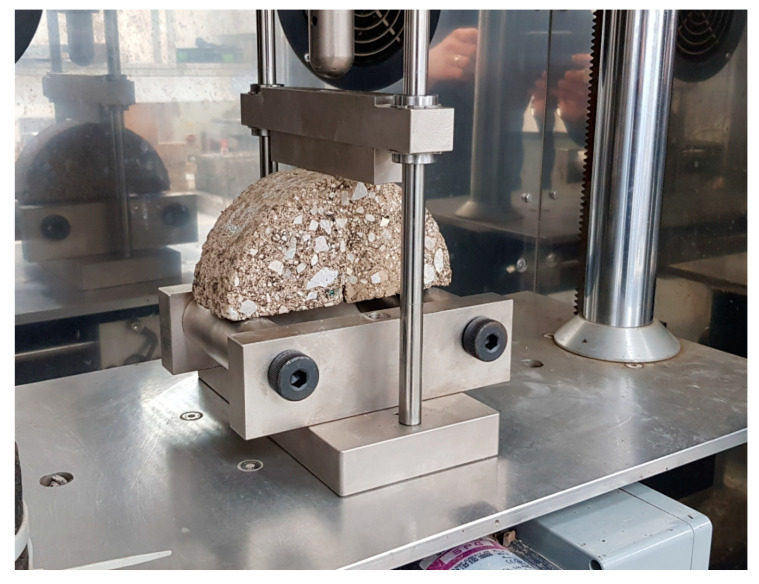
Semi-circular bending test set-up and specimen, fracture toughness test set-up (laboratory at Faculty of Civil Engineering and Architecture, Kielce University of Technology, Kielce, Poland) (source: M. Krasowski).

The resistance of the material to crack propagation was revealed using a semi-circular specimen with a notch N cut in the base of the specimen. The notch parameters 1.0 ± 0.10 mm in nominal width and 10.0 ± 1.0 mm in length were chosen according to Szydłowski [4]. The specimen was subjected to a three-point bending test. The central part of the base side of the specimen was subjected to tensile stress increasing at a constant deformation rate of 5 mm/min. The corresponding load was increased to the maximum value *F_max_*, which is directly related to the fracture toughness of the specimen. Figure 4 shows an example of the bend test set-up and the test specimen.

Test temperature was selected to be 0 °C. Before testing, the specimens were stored in an environmental chamber for at least 4 h at 0 ± 1 °C. The maximum strain, *ε_max_*, was calculated as follows:

(9) $$\varepsilon_{max,i}=\frac{\Delta W_{i}}{W_{i}}\times 100\%$$

where *W_i_* is the height of specimen *i* (*i* = 1, 2, 3, 4) (mm) and ∆*W_i_* is the vertical strain at maximum force/load on specimen *i* (*i* = 1, 2, 3, 4) in (mm).

The maximum peak stress/the critical stress, *σ_max_*_, *i*,_ was calculated as follows:

(10) $$\sigma_{max,i}=\frac{F_{max,i}}{D_{i}\cdot t_{i}}N/{\mathrm{mm}}^{2}$$

where *D_i_* is the diameter of specimen *i* (*i* = 1, 2, 3, 4) in (mm), *t_i_* is the thickness of specimen *i* (*i* = 1, 2, 3, 4) in (mm), and *F_max_*_,*i*_ is the maximum load on specimen *i* (*i* = 1, 2, 3, 4) in (N).

The fracture toughness, *K_IC_*, of specimen *i* (*i* = 1, 2, 3, 4) was calculated as follows:

(11) $$K_{Ic,i}=\sigma_{max,i}\cdot Y\;\cdot \;\sqrt{\pi \cdot a_{1}}\;N/{\mathrm{mm}}^{3/2}$$

(12) $$Y=4.782-1.219\cdot \left(\frac{a_{i}}{r_{i}}\right)+0.063exp\left(7.045\cdot \left(\frac{a_{i}}{r_{i}}\right)\right)$$

where *a_i_* is the length of the crack in specimen *i* (*i* = 1, 2, 3, 4) (mm), *σ_max_*_,*i*_ is the critical stress on specimen *i* (*i* = 1, 2, 3, 4) (MPa), and *Y* is the normalized stress factor by Formula (12).

It should be emphasized that the assessment of CBM resistance to crack propagation is essential for the durability of road pavements. Fatigue cracks that appear in the cement-bound mixtures are transferred through the upper layers and reflect on the surface. It thus seems warranted to search for solutions capable of increasing the fracture toughness of CBM.

## 5. Mixture Design and Test Specimen Preparation

### 5.1. Mixture Design

**Figure 5:**
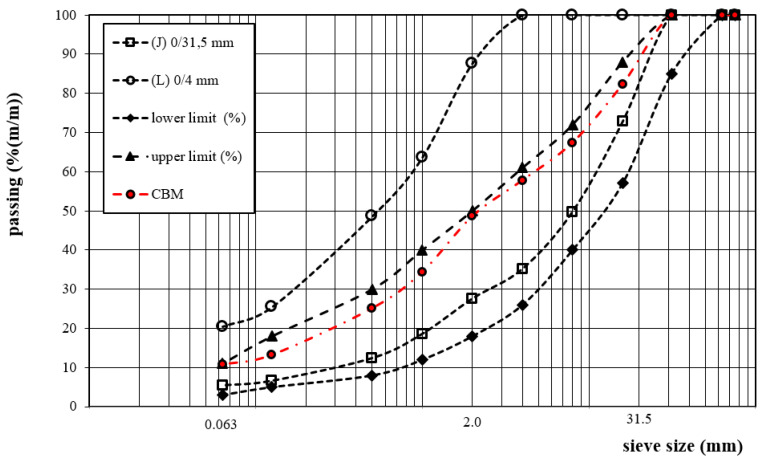
Grading curve of the CBM and mineral aggregate.

The mineral mix design consisted of determining the proportions of individual components to meet the optimal particle size distribution criterion limited by the lower and upper particle size distribution curve. The optimal particle size distribution area of CBM with a particle size up to 31.5 mm was used following the guidelines set forth in [33]. The mineral mixture grading curve’s optimal plot was obtained for the following proportions: 80% aggregate (J) with a grain size of 0/31.5 mm and 20% aggregate (L) with a grain size of 0/4 mm. The particle size distribution curve is shown in Figure 5.

The percentage content of particular fractions in the mixture was as follows: 10.7% filler (≤0.063 mm), 37.9% sand (0.063–2.0 mm), and 51.4% grit (≥2 mm).

### 5.2. CBGM Preparation and Curing

The test mixtures were prepared under laboratory conditions. A WLB10 laboratory mixer was used to obtain a homogeneous mixture. The single batch size was 35 kg. Before testing, it was necessary to determine the optimal amount of water in the mineral mixture (OMC (Optimum Moisture Content)) as per the guidelines outlined in EN 13286-2 [56] following the Proctor method. The value of OMC was 7.0%.

The specimens were compacted in the laboratory according to the requirements specified in the test method. The basic physical and mechanical properties (i.e., bulk density, water absorption, void content, indirect tensile strength, and stiffness modulus) were determined on the specimens prepared using a Marshall hammer and dedicated perforated molds [57,58]. The process consisted of applying 75 blows per side of the specimen at 60 blows per minute.

In the case of the axial compressive strength (R_C_) test, the specimens were prepared as per the standard [56] in B-type Proctor molds and compacted following the requirements of the modified Proctor method.

The crack resistance was determined in the same way as for bituminous mixtures, i.e., in a semi-circular bend (SCB) test. The samples were compacted using a gyratory compacting press [4,53]. The settings of the compactor were adopted based on the literature data and the authors’ experience. The selected number of revolutions provided the maximum volumetric density at which the void content in CBM was equal to *V_m_* = 10.0%.

On the first day, the so-prepared specimens were stored at room temperature + 20 ± 5 °C in the molds. After demolding, the specimens were stored for 27 days at 40–70% humidity. The tests started after a conditioning period of 28 days. The same testing procedure was used regardless of the compaction method applied.

## 6. The Microstructure of Cement–Polymer Composite

The polymer–cement binder was obtained by mixing Portland cement with polymer powder. The role of polymer particles in the cement-bound mixture is very similar to that in cement concrete. First, they are dispersed in the liquid phase of the cement paste [27,59,60]. Once the hydration process starts, the polymer appears on the cement grains and aggregates’ surface. The dispersed polymer particles are also trapped in the capillary pores of the aggregate, where after the evaporation of water they form dense agglomerates.

Along with the drying of the mixture, the polymer coalesces, i.e., individual particles stick together and form a continuous polymer film. A schematic diagram of the formation of the microstructure described based on the literature data [27,59,60].

Modification of concrete mixtures with polymers offers many advantages [27]. One of the benefits is a noticeable increase in water tightness and workability. Mixtures made with polymers have a lower stiffness, which translates to higher tensile and flexural strengths. Improved adhesion to aggregate particles increases the cohesion of the mixture.

**Figure 6:**
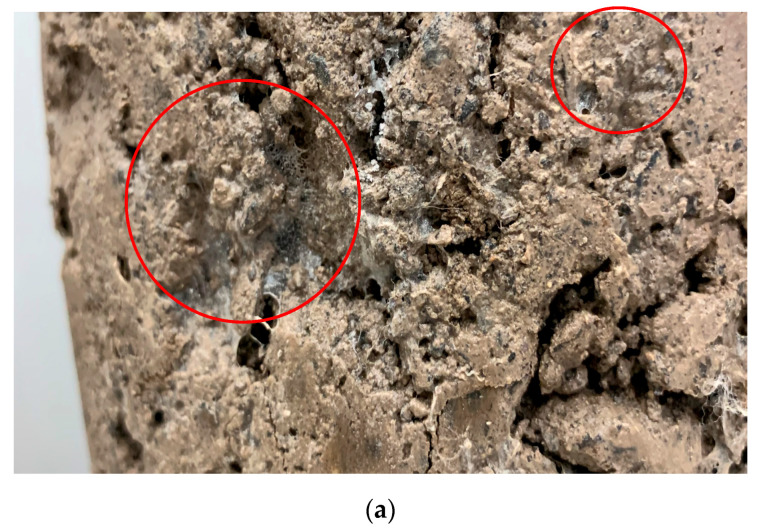

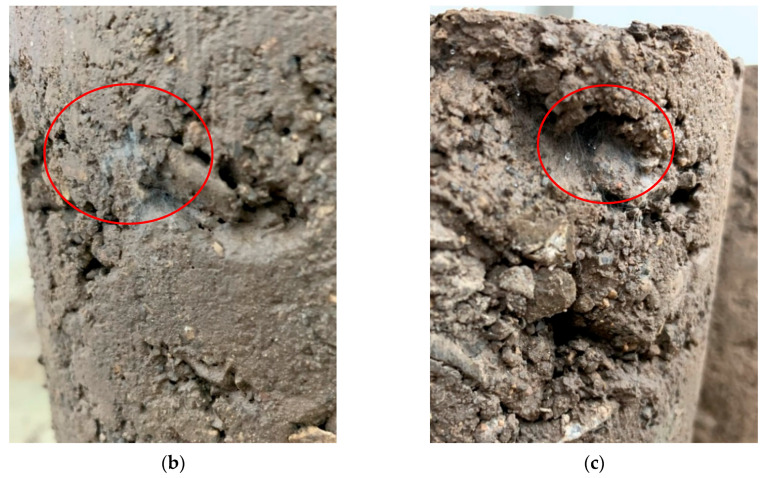
Visible polymer film on the surface of CBM grains. (**a**) CBM_C2P4; (**b**) CBM_C0P2; (**c**) CBM_C2P2, (source: M. Krasowski).

The polymer film formed on the surface of the lab-made CBM confirms the adopted premise about the use of polymer powder in CBM mixtures. As shown in Figure 6, the polymer powder causes single particles of the mixture to coalesce even when there is a void space between them (Figure 6a). The bridging of the void spaces between the aggregate particles (red circles in Figure 6a–c) is a characteristic feature of the continuous polymer layer. This ability results from excellent adhesion between the polymer and mineral surfaces in the cement-mineral mixture [27].

## 7. Results and Analysis

The effect of the RPP and CEM contents was represented by the models developed based on Formula (2). The parameters were consistent with the adopted testing methodology described in Section 4.

Physical and mechanical properties:

- Bulk density—saturated surface dry (SSD) (*ρ_bssd_*) [48]
- Water absorption by weight (*n_w_*) [49]
- Elastic modulus in the IT-CY test (*S_m_*) [50]
- Compressive strength at 28 days (*R_C_*) [51]
- Indirect tensile strength (*ITS_DRY_*) [52]

Resistance to cracking [61]:

- Maximum strain (*ε_max_*) [53]
- Critical stress (*σ_max_*) [53]
- Fracture toughness of the mixture (*K_IC_*) [53]

STATISTICA software was used to estimate a regression model describing the relationship between the factors (RPP content and CEM content) and the parameters under analysis (*ρ_bssd_*, *V_m_*, *n_w_*, *S_m_*, *R_C_, ITS_DRY_*, *ε_max_*, *σ_max_*, and *K_IC_*). In Table 8, Table 9, Table 10, Table 11, Table 12, Table 13 and Table 14, the significant influence of CEM and RPP contents on the given parameter is marked in red.

### 7.1. Regression Models in the Response Surface Methodology for Changes in Physical and Mechanical Properties of CBM in Terms of the CEM Content and the RPP Content

**Figure 7:**
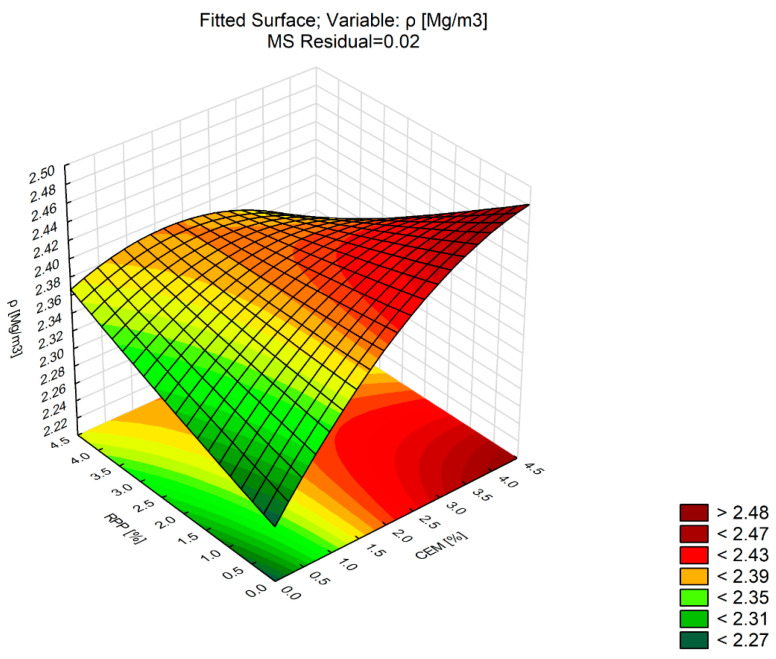
Response surface plot of *ρ_bssd_* versus RPP and CEM (CBM mixture).

Density is the essential parameter that characterizes building materials. It is used to determine to what extent the developed mix composition affects the internal structure of the material. The density of a composite consisting of materials with different densities (CEM and RPP) is influenced by the content of the component having higher density. Table 8 shows the influence of the factors (CEM and RPP) and the interaction between them on bulk density (*ρ_bssd_*). The regression model of the effect of polymer powder and Portland cement on the bulk density of the bound mixture is shown graphically in Figure 7.

Analysis of the statistical parameters (Table 8) shows that the CEM content and the interaction between CEM and RPP factors have a significant effect on the density (*p*-Value < 0.05). The value of the R^2^ statistic obtained for *ρ_bssd_* indicates a very good fit of the second-order model to the test results, as R^2^ = 0.95 indicates that the polynomial explains 95% of the variability of the characteristic analyzed.

Note that the CBM mixture density level increased mainly with the increase in Portland cement content. In the cement dosage range of 2–4%, the rapid density increase was due to the increased proportion of Portland cement, i.e., the component with the highest density. The RPP content did not affect CBM density. At the maximum CEM content of 4%, the bulk density of the CBM decreased with the increasing amount of polymer powder. At C0P0, the density of the mixture refers to the bulk density of the mineral matrix and results from the regression model description.

Another physical property under analysis was water absorption (*n_w_*). After building a mathematical model of regression (response surface) to analyze the effect of the RPP content and the CEM content on water absorption variability, statistical significance of the model fitting parameters was determined.

**Figure 8:**
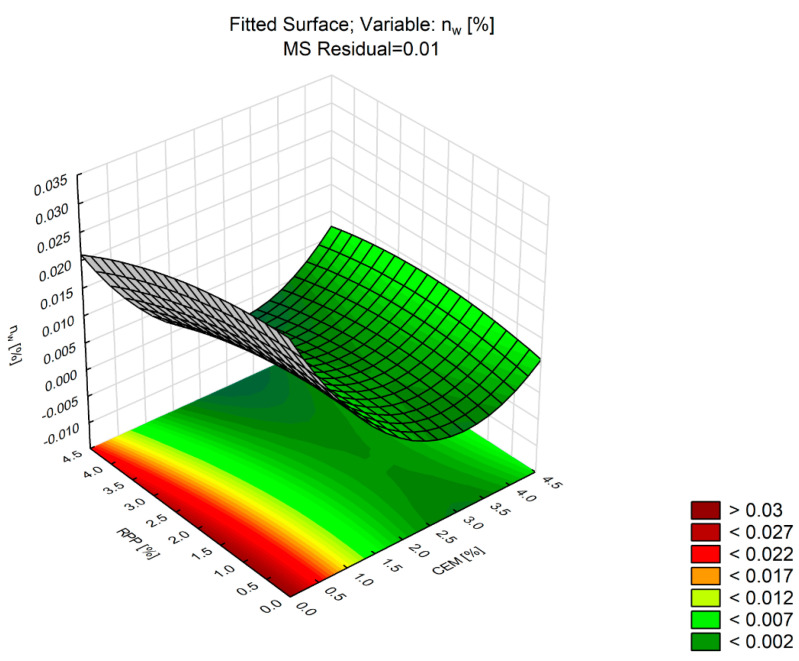
Response surface plot of *n_w_* versus RPP and CEM (CBM mixture).

Table 9 shows the impact of the factors on the values of water absorption *n_w_*. Figure 8 shows a graphical representation of the model.

Analysis of the response surface for water absorption (*n_w_*) shows that the fit value of the second-order model explains 97% of the *n_w_* variability. The correlation coefficient is 0.97.

The data in Table 9 show significant linear and nonlinear effects of the CEM factor. A significant nonlinear influence of RPP content (RPP [%] (Q)) was observed. Besides, the effect of the CEM factor (*p*-Value <0.0001) was greater than that of the RPP factor (*p*-Value = 0.007). The response surface shown in Figure 8 confirms the influence of these factors on changes in *n_w_*.

An increase in CEM content within the 2–4% dosing range did not induce any significant change in water absorption values. Regardless of the RPP amount used, the values for that range were below 0.5%. The highest water absorption was observed in the absence of Portland cement in the mixture. The water absorption values for the mixtures designated as C0P2 and C0P4 were higher than 2.5%. These relationships do not yield beneficial results from a durability point of view, although the water absorption values are, for this material, at a safe level of less than 5% [33].

**Figure 9:**
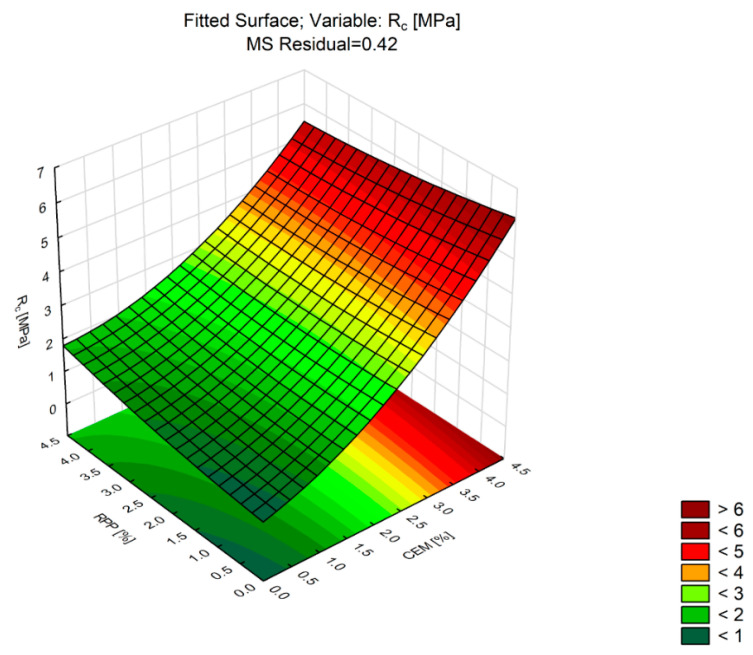
Response surface plot of *R_c_* versus RPP and CEM (CBM mixture).

The axial compressive strength (*R_C_*) test is the basic criterion for assessing mineral–cement composites [62,63]. The impact of the factors on the change of axial compressive strength *R_c_* was determined on eight sets of samples, supplemented with a minimum of three replicates, varied in the Portland cement content and polymer powder amount. Table 10 summarizes the analysis, while Figure 9 shows the response surface described by the second-degree polynomial.

Analysis of the results in Table 10 shows a significant influence of CEM content on the axial compression strength *Rc* at the assumed significance level, *p*-Value = 0.05. No effect of RPP was found. The Portland cement content affected the CBM’s response. The change in axial compression strength after 28 days was nonlinear relative to the amount of Portland cement. The highest strength value of more than 4.5 MPa was recorded at the maximum CEM content (4%). Analysis of the effect of the RPP content on compressive strength at the CEM content of 4% shows that the compressive strength values remained at the similar level. A slight decrease was observed as the RPP content increased. This relationship occurred at each point of the Portland cement dosage range. The lowest 28-day axial compression strength (*R_C_*) was found when only the polymer powder was present in the mixture. Increasing the amount of polymer powder from 2% to 4% increased the strength from 0.7 to 2.0 MPa. The increase in cement amount from 2% to 4% increased the strength from about 2.50 to 5.10 MPa. Strength increase increments for both binders were similar, and the order of magnitude more than doubled.

**Figure 10:**
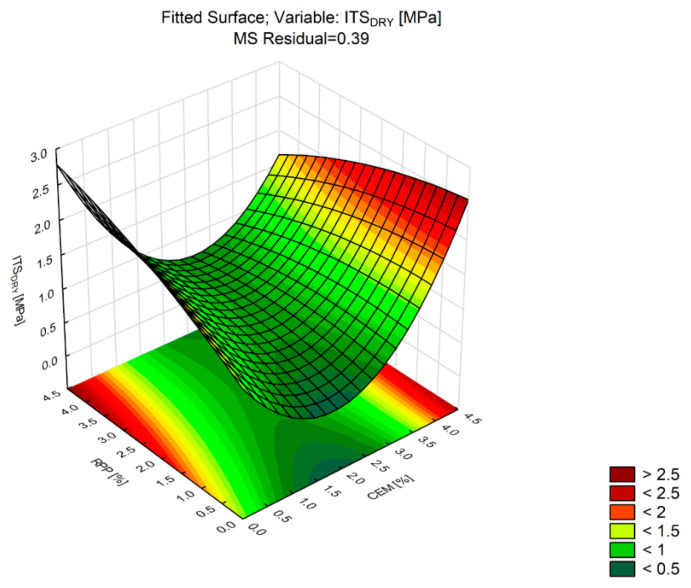
Response surface of *ITS_DRY_* versus RPP and CEM (CBM mixture).

Indirect tensile strength (*ITS_DRY_*) is the most important parameter for the durability of pavements. Base courses made with bituminous and cement binders experience a range of tensile stresses [28,64]. Appropriate values of indirect tensile strength (*ITS_DRY_*) ensure durable pavement structures [65,66,67,68]. The test was performed according to the testing plan in Section 4 on eight sets of samples supplemented with a minimum of three replicates, varied by levels of Portland cement and polymer powder contents. Table 11 summarizes the analysis, while Figure 10 shows the response surface described by the second-degree polynomial.

Analysis of the statistical parameters in Table 11 shows that at the adopted significance level *p*-Value = 0.05, the indirect tensile strength variable (*ITS_DRY_*) is mostly affected by the content of CEM (*p*-Value = 0.000). The change in the *ITS_DRY_* value due to the RPP content has a linear plot. The value of R^2^ indicates a very good fit of the polynomial to the test results. As R^2^ = 0.98, the polynomial explains 98% of the *ITS_DRY_* variability. The presence of RPP in CBM changes the *ITS_DRY_* parameter to a degree similar to that obtained at the maximum CEM concentration (4%). Unlike in the case of axial compression strength (*R_c_*), the *ITS_DRY_* values for mixtures C0P2 and C0P4 were higher than those for mixtures C2P0 and C4P0. This indicates that it is possible to obtain a higher indirect tensile strength of CBM without Portland cement in the mixture. The highest strength, *ITS_DRY_* = 2.75 MPa, was observed in mixture C0P4, and the lowest strength, *ITS_DRY_* = 0.45 MPa, was observed in mixture C2P0. An interesting relationship was observed at maximum concentrations of Portland cement and polymer powder in the mixture (CBM_C4P4), in which the indirect tensile strength was found to be lower than that of the mixtures without Portland cement. Compared to the indirect tensile strength value recorded for C0P4, the *ITS_DRY_* for C4P4 and C4P2 was 120% and 65% lower, respectively. These relationships lead to the conclusion that the polymer powder in the CBM mixture can be used to reduce axial compressive strength (Figure 9) while maintaining high indirect tensile strength (Figure 10).

**Figure 11:**
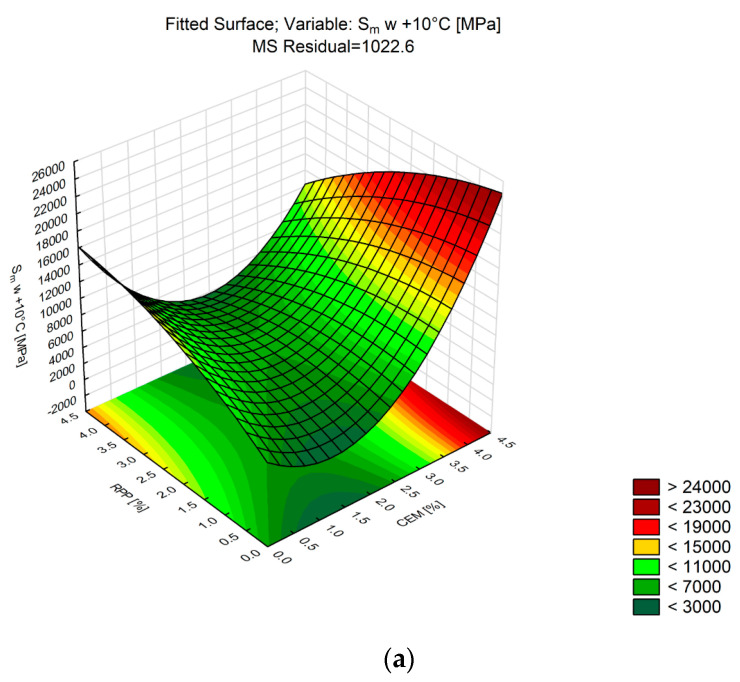

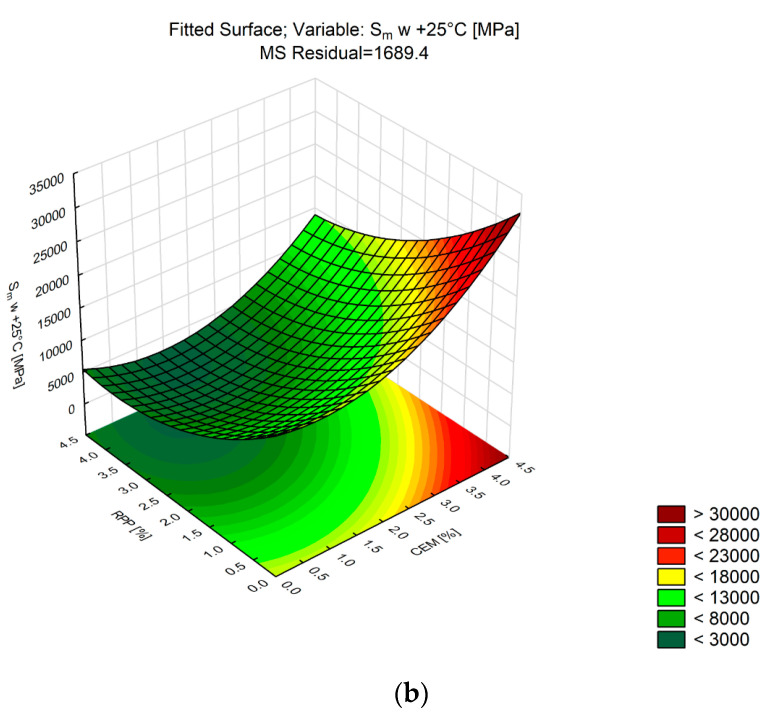
Response surface plots of *S_m_* versus RPP and CEM (CBM): (**a**) at +10 °C; and (**b**) at +25 °C.

Stiffness modulus (*S_m_*) is an important parameter used in the design of the pavement layer system [69]. To determine the stiffness modulus of CBM mixtures, the indirect tensile test on cylindrical specimens (IT-CY mode) was performed at +10 and +25 °C, following the methodology described in Section 4. The analysis is summarized in Table 12. Figure 11 shows the response surface.

The correlation coefficient variability ranged from 0.96 to 0.98, which means that the nature of changes in CBM stiffness modulus was explained to a similar degree. Analysis of the data in Table 12 indicates that the effects of both the CEM content and the RPP content were significant but showed different trends with the change in test temperature. At the lower temperature (+10 °C), both factors showed statistically significant linear and nonlinear effects at the significance level α = 0.05. The maximum values of the stiffness modulus were recorded for C4P4 containing maximum amounts of cement and polymer. The minimum values of the modulus were not reached for this combination. At +10 °C, the minimum value was obtained for C2P0, and at +25 °C for C0P4. It can thus be stated that the incorporation of polymer powder into the composition of the cement-bound mixture makes the mixture more sensitive to temperature changes. This observation is confirmed in the analysis of changes in stiffness modulus at the maximum amount of Portland cement and the gradual increase in the amount of polymer powder from 0% to 4%. Regardless of test temperature, the increase in the amount of polymer powder causes a decrease in stiffness modulus. Similar relationships for stiffness modulus (S_m_) occur in bituminous mixtures [70], where the increase in bitumen quantity and temperature reduces the value of stiffness modulus.

### 7.2. Regression Models in Response Surface Methodology for Changes in Fracture Toughness (K_IC_) in Terms of the Amount of Portland Cement and the Amount of Polymer Powder

Fracture toughness of cement-bound mixtures is important from the perspective of pavement design. Determining the fracture mechanics parameters of cement-bound mixtures based on bending of notched semi-cylindrical specimens is a relatively simple and quick task [61]. The test system used to evaluate the fracture toughness of CBM was the same as that for evaluating the fracture toughness of bituminous mixtures [71,72]. Fracture toughness is analyzed by looking at the critical stress intensity factor, *K_IC_*, which is used in the calculation of pavement fatigue life in accordance with the guidelines set out in [73]. To determine the fracture toughness described by Formula (11), it was necessary to determine the maximum strain *ε_max_* and the maximum stress at failure *σ_max_* in the tests. The results of these tests are compiled in Table 13.

Analysis of the results in Table 12 indicates that the presence of polymer powder in the cement-bound mixture (CBM) contributes to greater strain with a concurrent increase in stress at the failure of the mixture. Comparison of the extreme cases, i.e., the mixtures containing Portland cement and no polymer (C2P0 and C4P0) and those containing the polymer powder and no Portland cement (C0P2 and C0P4) shows that a 2% increase in cement amount causes an 18% increase in strain, while with the polymer powder content increased by 2%, the strain at failure increases by 81%. These relationships prove that the base layers made with polymer powder are more flexible at higher stress at failure. This is indicative of higher resistance under loading.

**Figure 12:**
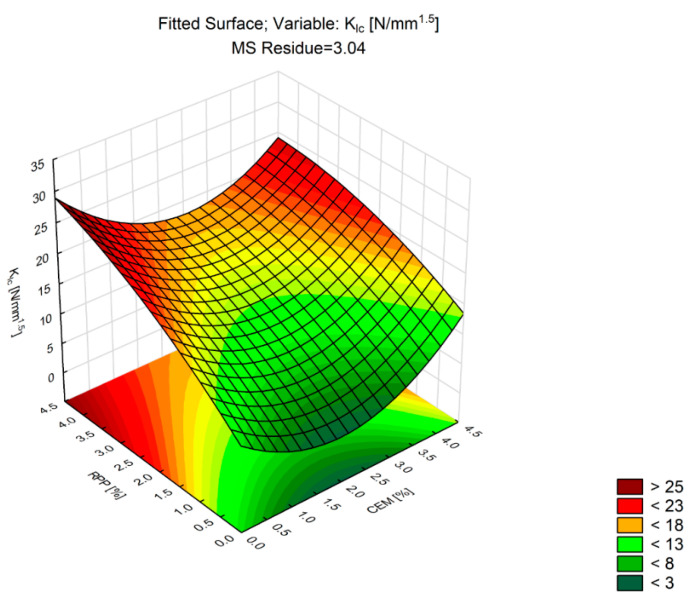
Response surface plot of ***K_IC_*** versus RPP and CEM (CBM).

A comprehensive evaluation of fracture toughness was performed by analyzing the *K_IC_* factor in terms of CEM and RPP amounts in CBM mixtures. The results of the analysis are given in Table 13. Figure 12 shows a graphical representation of the model.

As it follows from the results in Table 14, the amount of Portland cement (CEM) and the amount of polymer powder (RPP) have significant effects on fracture toughness (*K_IC_*) of the bound mixture at the assumed significance level, *p*-Value = 0.05. The mixtures containing polymer powder show the highest fracture toughness (*K_IC_*), with the top value, 28.79 N/mm^3/4^, attained by the mixture made with 4% polymer powder and no cement. A reduction in polymer powder content reduces the resistance to 2.87 N/mm^3/4^, as obtained by the mixture containing Portland cement at 2%. Note that the rate of the parameter variations increases with the increase in the amount of polymer powder, regardless of the cement content.

Overall, the analysis of the selected parameters against the content of Portland cement (CEM) and the content of polymer powder (RPP) allowed constructing the models of regression shown in Table 15.

The results in Table 15 indicate a significant effect of polymer on nearly all properties, independently or through interactions. The polymer powder had a statistically insignificant influence on *R_c_* variability, as opposed to the cement, which was exclusively responsible for building the elastic–brittle matrix. The polymer effect would have been more pronounced if the specimens had been compressed at the stress rate increment less than 0.5 MPa/s. The determinations of *ITS_DRY_* and *K_IC_*, which achieve the limit state at tensile stress, indicate that the addition of RPP is necessary as the base layer is mainly affected by fatigue damage (tensile stresses in the lower part of the bound base layer). The strong correlation between RPP and the properties relating to crack formation indicates the need for tRPP and CEM content optimization.

## 8. Determination of the Recommended Percentage Amounts of Portland Cement and Polymer Powder to Increase the Fracture Toughness of the Bound Mixture. Optimization by the Desirability Function Method

**Figure 13:**
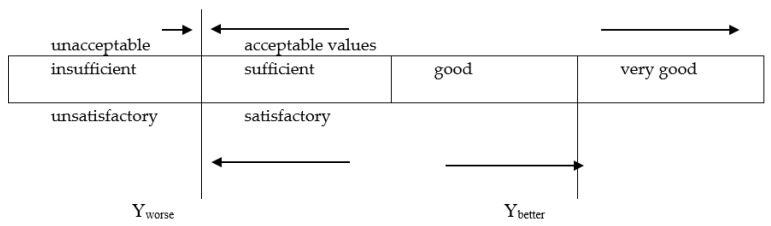
Desirability criterion scale [47].

Comprehensive evaluation of the materials against all criteria requires applying multi-criteria statistical optimization using the generalized desirability function with one-sided transformation [47]. In this method, the values of the criteria are expressed on a common dimensionless scale. The construction of such a scale requires determining the range of satisfactory values for each criterion, with the indication of the inferior value *y_G_^(i)^* and the superior value *y_L_^(i)^.* If greater values for the test variable are preferred, then *y_G_^(i)^* < *y_L_^(i)^*; if smaller values are preferred, then *y_G_^(i)^* > *y_L_^(i)^.* Figure 13 shows the schematic diagram of the desirability criterion.

In addition to ranges of satisfactory values, weights are assigned to the individual criteria. The sum of the weights must be equal to 1. The adoption of ranges of satisfactory values and weights for particular features is based on the knowledge of technical requirements in the intended use of the material.

Utilities *U_i_^III^* are numbers within the 0 to 1 interval with those close to 0 representing completely undesirable values of *y^(i)^* and those close to 1 representing the most desirable values.

*y^(i)^* values in the range 0.37–0.63 are regarded as satisfactory. In this range, the desirability function is most sensitive to changes in the values of a given variable. The relevant intervals are shown in Table 16.

Harrington [75] proposed the expression for the generalized profile of the desirability function (13):

(13) $$\begin{array}{c} U^{III}=\exp\left[-\exp\sum_{i=1}^{m}w_{i}\cdot \exp\left(-\frac{y^{\left(i\right)}-y^{\left(i\right)}{}_{G}}{y_{L}{}^{\left(i\right)}-y_{G}{}^{\left(i\right)}}\right)\right]\\ 0\;\leq \;w_{i}\;\leq \;1;\;i\;=\;1,\;2,\ldots m;\;\sum_{i=1}^{m}w_{i}=1 \end{array}$$

The value of utility *U^III^* is markedly reduced if at least one of the criteria assumes a non-acceptable value (worse than satisfactory). This raises a chance for all criteria to take on at least satisfactory values at the point corresponding to the maximum utility *U^III^* [46].

Determining the value of the desirability function allows the assessment of the tested material based on the entire set of criteria adopted, with their validity taken into account. Optimization with a desirability approach starts with multiple criteria. In the final phase, those criteria are combined into a single, complex criterion that integrates all the requirements in one indicator.

Analysis of the dependence of generalized utility on the considered factors enables the determination of such combinations of factor values for which one can expect particularly beneficial material properties. The determination of the desirability function for individual material types allows ranking them according to their suitability for a given application.

The optimal solution is the result of the optimization process based on the adopted criteria. Changing the criterion significantly affects the result of desired output estimation in terms of material properties. Therefore, the possibility of obtaining such a solution was considered in terms of the amount of Portland cement and the amount of polymer powder, the combination of which would allow obtaining high fracture toughness (*K_IC_*) of the mixture (CBM), and the optimization result would be at least acceptable (Table 15).

Table 17 lists the optimization criteria.

Table 16 includes numerically defined values either adopted from the literature and standards or derived from knowledge of the particular field. The “min” and “max” values were adopted based on the results obtained from this study, without any literature-confirmed criteria. This procedure was to eliminate the possibility of imparting desirability function values 0 or 1 to the results obtained for the bound mixtures.

To select the mixtures (CBM) demonstrating a higher resistance to cracking, the *K_IC_* factor adopted for optimization was the same as for bituminous mixtures of high fracture toughness [72]. To highlight the flexible character of the bound mixtures, the stiffness modulus (*S_m_*) and the axial compressive strength (*R_C_*) were minimized. The optimization criterion of cohesion described by the indirect tensile strength (*ITS_DRY_*) was defined such that the mixtures with ITS_DRY_ above 0.50 MPa were the most desirable. This constraint allowed selecting the mixtures with low stiffness but high crack resistance. Note that obtaining such conflicting relationships is impossible in the case of conventional bituminous mixtures and cement-bound mixtures, where the relationship is inversely linear and an increase in strain leads to a decrease in crack resistance.

**Figure 14:**
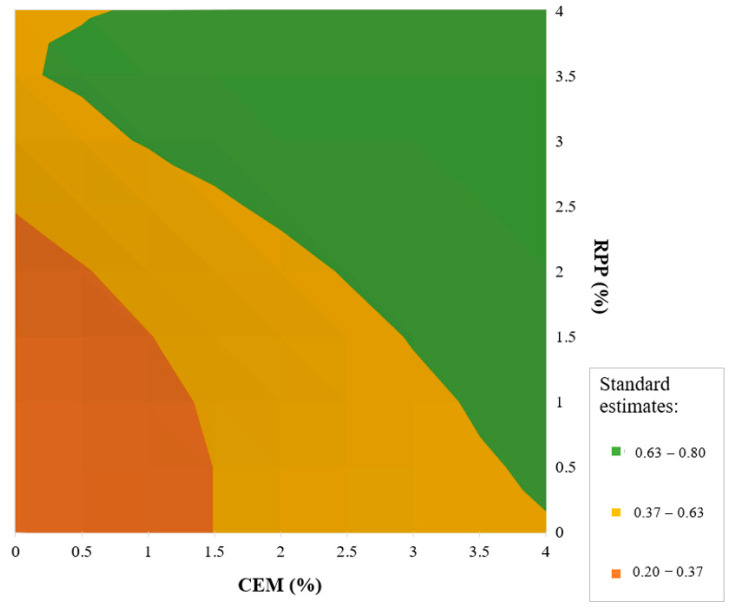
Response surface for the desirability function of the CMB mixture.

Optimization was performed based on previously estimated regression models (Table 14). Optimization results have a step of 0.5% representing the amount of a given binder. It was possible because the results approximated from the models are optimized. Figure 14 shows the results of the optimization for the bound mixture (CBM).

The set of values representing a good quality of fitting the desirability function, *U^III^* ϵ (0.63–0.80), according to the optimization criteria (Table 16) for mixtures with higher fracture toughness is marked in green (Figure 14). The set of solutions includes bound mixtures containing polymer powder over the entire dosing range. Increasing the amount of polymer powder allows reducing the amount of Portland cement. The reduction of polymer powder amount in the mixture forces the use of an increased amount of Portland cement. The use of a small amount of polymer powder, above 0.5%, with the maximum concentration of Portland cement increases the crack resistance of cement-bound mixtures. In this experiment, the binder content of 4% (CEM + RPP) ensured a high-quality base layer. Satisfactory effectiveness of this mixture can be obtained by adding each component at 2% or more as *U^III^* > 0.37 (Table 16). This solution is suitable for designing cement-bound mixtures with this binder type. The unacceptable result, *U^III^* ϵ (0.20–0.37), occurs when the composition of the bound mixture contains both components in the amount from 0% to about 1.5% in varying proportions. This result is marked in orange. The use of components (RPP and CEM) in the proportion from 0% to about 1.5% does not guarantee the resistance to cracking for the set mixture.

Attention should be paid to possible interactions when designing a binder from a mixture of cement and polymer. The use of one of the components alone does not guarantee the same efficiency in providing general durability of the cement-bound mixture. Note that the efficacy of the binder increases when the content of each component rises proportionally.

## 9. Conclusions

Based on the tests and analyses of cement-bound mixtures modified with polymer powder, the following conclusions were reached:

- The beneficial effect of the polymer powder on the crack resistance of cement-bound mixtures (CBMs) was confirmed.
- Macroscopic analysis of the samples of mixtures bound with cement and polymer powder revealed bridging between aggregate grains after the conditioning period; this confirms the formation of a continuous polymer phase and modification of the structure of the cement-bound mixture.
- No effect of the polymer powder (RPP) on the change in the CBM absorption parameter was found. However, it was found that an increase in the amount of cement within the range 2–4% reduced this parameter.
- No change in compressive strength of the cement-bound mixture was observed when the amount of polymer powder was changed. The increase in strength was related to the change of the CEM amount. Increasing the content of polymer powder within the range 2–4% increased the strength from 0.7 to 2.0 MPa. On the other hand, increasing the CEM amount within the range 2–4% increased the strength from about 2.50 to 5.10 MPa. The strength increments for both binders are similar, while the order of magnitude more than doubles when using cement.
- Polymer powder in the bound mixture reduces axial compressive strength while maintaining high resistance to indirect tensile strength.
- The use of polymer powder at more than 0.5% with a maximum concentration of Portland cement (4.0%) has a positive effect on the fracture toughness of CBM.
- The mixtures containing polymer powder are characterized by much higher fracture toughness than the twin combinations with Portland cement.

## Figures and Tables

**Table 1:** Characteristics of base layer bound mixtures.

Reference	Reclaimed Asphalt Pavement (RAP) Content (%)	Mineral Aggregate Content (%)	Hydraulic Binder Content and Type (%)	Bitumen Binder Content and Type (%)	Others Binder Type (%)
Dołżycki et al. [19]	70%	30%	2%/4%/6%—cement	2/4/6%—bitumen emulsion	-
Valentin et al. [20]	60–90%	10–40%	1.5–3.0%—cement	2.5%—bitumen emulsion	Waste filler
Buczyński et al. [21]	50%	50%	2%—cement	2.4%—foamed bitumen (50/70)	5%/12.5%/20% Dolomite Dust
Graziani et al. [22]	50%/80%/0%	50%/20%/100%	2%—cement	3.0%—bitumen emulsion	-
Lin et al. [23]	100%		2%—cement	4.0%—bitumen emulsion	-
Skotnicki et al. [24]			2.38%, 3.38%, 4.38%—cement	3.5%, 5.5%—foamed bitumen	-
Niazi et al. [25]	20%	80%	0%, 0.5%, 1.0%, 2.0%—cement	3.5% emulsion	0%, 0.5%, 1.0%, 2.0% lime

**Table 2:** Polymer EVA components [32].

Component	Percentage (%) (m/m)
Vinyl acetate (VAc)	70.0–100.0
Copolymer (butyl acrylate. ethylene. edetic acid vinyl ester)	0.0–30.0
partially hydrolyzed poly (vinyl alcohol)	6.0
sodium bicarbonate	0.3
hydrogen peroxide (35%)	0.7
sodium hydroxymethyl sulfonate	0.5
water	80.0

**Table 3:** Chemical composition of the redispersible polymer powder used in this study.

Component	Percentage in EVA (%)
C	67.67
O	29.13
Mg	0.52
Si	1.65
Ca	0.75
Al	0.29
∑	100.00

**Table 4:** Properties of dolomite aggregates 0/31.5 (J).

Property	Test	u.m.	Result	Symbol
Dimension d/D	EN 933-1 [34]	-	-	0/31
Particle size distribution	EN 933-1 [34]	-	-	GA_85_
Density	EN 1097-6 [35]	Mg/m^3^	2.71	2.71
Shape index	EN 933-4 [36]	%	16.0	SI_20_
Flakiness index	EN 933-3 [37]	%	14.0	FI_20_
Percentage of crushed and broken surfaces	EN 933-5 [38]	%	98/2	C_90/3_
Frost resistance	EN 1367-1 [39]	%	3.4	F_1_
Resistance to fragmentation	EN 1097-2 [40]	%	23	LA_30_
Abrasion resistance	EN 1097-1 [41]	%	17.5	M_DE_25

**Table 5:** Properties of dolomite aggregate 0/4 (L).

Property	Test	u.m.	Result	Symbol
Dimension d/D	EN 933-1 [34]	-	-	0/4
Particle size distribution	EN 933-1 [34]	-	-	G_F_85
Density	EN 1097-6 [35]	Mg/m^3^	2.83	2.83

**Table 6:** Properties of Portland cement CEM I 42.5R.

Property	Test Method	Unit of Measure	Result
Initial setting time	EN 196-3 [43]	min	209
Compressive strength	EN 196-1 [44]		
at 2 days		MPa	27.2
at 28 days		MPa	55.6
Soundness	EN 196-3 [43]	mm	0.8
Specific surface area	EN 196-6 [45]	cm^2^/g	3360

**Table 7:** Design of experiment.

Property	Portland Cement Content (%)	Polymer Powder Content (%)
CBM_C2P0	2	0
CBM_C4P0	4	0
CBM_C2P2	2	2
CBM_C2P4	2	4
CBM_C4P2	4	2
CBM_C4P4	4	4
CBM_C0P2	0	2
CBM_C0P4	0	4
CBM_C0P0	0	0

**Table 8:** Experimental coefficients for bulk density *ρ_bssd_*_._

Factor	Regr. Coefficients *ρ_bssd_* [Mg/m^3^] R-sqr = 0.95; MS Residual = 0.02	
	Regression Coefficient	*p*-Value
Mean/Interc.	2.26	0.001
(1) CEM [%] (L)	0.087	0.001
CEM [%] (Q)	−0.008	0.022
(2) RPP [%] (L)	0.021	0.335
RPP [%] (Q)	0.001	0.867
1L by 2L	−0.013	0.005

Q, quadratic; L, linear.

**Table 9:** Experimental coefficients for water absorption *n_w._*

Factor	Regr. Coefficients *n_w_* [%] R-sqr = 0.97; MS Residual = 0.01	
	Regression Coefficient	*p*-Value
Mean/Interc.	0.031	0.001
(1) CEM [%] (L)	−0.020	0.001
CEM [%] (Q)	0.003	0.001
(2) RPP [%] (L)	−0.001	0.950
RPP [%] (Q)	−0.001	0.001
1L by 2L	0.001	0.001

Q, quadratic; L, linear.

**Table 10:** Experimental coefficients for axial compressive strength *R_c._*

Factor	Regr. Coefficients *R_C_* 28d [MPa] R-sqr = 0.94; MS Residual = 0.42;	
	Regression Coefficient	*p*-Value
Mean/Interc.	0.780	0.254
(1) CEM [%] (L)	0.453	0.240
CEM [%] (Q)	0.166	0.021
(2) RPP [%] (L)	0.103	0.750
RPP [%] (Q)	0.026	0.638
1L by 2L	−0.084	0.158

Q, quadratic; L, linear.

**Table 11:** Experimental coefficients for indirect tensile strength *ITS_DRY._*

Factor	Regr. Coefficients *ITS_DRY_* [MPa] R-sqr = 0.98; MS Residual = 0.39	
	Regression Coefficient	*p*-Value
Mean/Interc.	1.380	0.001
(1) CEM [%] (L)	−1.065	0.001
CEM [%] (Q)	0.295	0.001
(2) RPP [%] (L)	0.448	0.004
RPP [%] (Q)	−0.030	0.132
1L by 2L	−0.115	0.001

Q, quadratic; L, linear.

**Table 12:** Experimental coefficients for stiffness modulus *S_m_* at +10 and +25 °C.

Factor	Regr. Coefficients *S_m_* at +10 °C [MPa] R-sqr = 0.98; MS Residual = 1022.6		Regr. Coefficients *S_m_* at +25 °C [MPa] R-sqr = 0.96; MS Residual = 1689.4	
	Regression Coefficient	*p*-Value	Regression Coefficient	*p*-Value
Mean/Interc.	6138.05	0.007	16272.79	0.001
(1) CEM [%] (L)	−5140.85	0.001	−1901.95	0.154
CEM [%] (Q)	2054.74	0.001	1214.75	0.001
(2) RPP [%] (L)	3965.11	0.001	−6058.27	0.010
RPP [%] (Q)	−384.44	0.023	812.55	0.014
1L by 2L	−1106.29	0.001	−375.63	0.190

Q, quadratic; L, linear.

**Table 13:** Average values of the results of fracture toughness determination.

Property	Portland Cement Amount (%)	Polymer Powder Amount (%)	Depth of Cut/Notch (mm)	∆*W* (mm)	*ε_max_* (%)	*σ_max_* (MPa)	*K_lC_* (N/mm^1,5^)
CBM_C2P0	2	0	10	0.37	0.50	0.11	2.87
CBM_C4P0	4	0	10	0.44	0.59	0.36	9.43
CBM_C2P2	2	2	10	0.60	0.83	0.52	13.90
CBM_C2P4	2	4	10	0.72	0.86	0.57	15.35
CBM_C4P2	4	2	10	0.69	0.90	0.60	16.07
CBM_C4P4	4	4	10	1.06	1.41	0.83	21.84
CBM_C0P2	0	2	10	0.73	0.97	0.72	19.23
CBM_C0P4	0	4	10	1.35	1.75	1.07	28.79

**Table 14:** Experimental coefficients for fracture toughness ***K_IC._***

Factor	Regr. Coefficients *K_IC_* (N/mm^1.5^) R-sqr = 0.91; MS Residual = 4.48	
	Regression Coefficient	*p*-Value
Mean/Interc.	9.31	0.002
(1) CEM [%] (L)	−6.74	0.001
CEM [%] (Q)	1.70	0.001
(2) RPP [%] (L)	6.42	0.001
RPP [%] (Q)	−0.45	0.073
1L by 2L	−0.44	0.057

Q, quadratic; L, linear.

**Table 15:** Experimental coefficients for the variables under analysis.

Property	Mean/Interc.	(1) CEM [%] (L)	CEM (%) (Q)	(2) RPP (%) (L)	RPP (%) (Q)	1L by 2L	R^2^
*ρ_bssd_*	2.26	0.087	−0.008	0.021	0.001	−0.013	0.95
*n_w_*	0.031	−0.020	0.003	−0.001	−0.001	0.001	0.97
*R_C_*	0.078	0.453	0.166	0.103	0.026	−0.084	0.94
*ITS_DRY_*	1.380	−1.065	0.295	0.448	−0.03	−0.115	0.98
*S_m_* + 10 °C	6138.05	−5140.85	2054.74	3965.11	−384.44	−1106.29	0.98
*S_m_* + 25 °C	1672.79	−1901.95	1214.75	−6058.27	812.55	−375.63	0.96
*K_IC_*	9.31	−6.74	1.7	6.42	−0.45	−0.44	0.91

**Table 16:** Qualitative evaluation of desirability function [46,74].

Standard Estimates	Desirability	Quality of Product
1.00	Excellent	Ultimate in “satisfaction” or quality; an improvement beyond this point would have no appreciable value
1.00–0.80	Very good	Acceptable and good. Represents an improvement over the best commercial quality, the latter having the value of 0.63
0.63–0.37	Satisfactory	Acceptable but poor. Quality is acceptable to the specification limits, but improvement is desired
0.37–0.20	Bad	Unacceptable. Materials of this quality would lead to failure of the project
0.20–0.00	Very bad	Completely unacceptable

**Table 17:** Optimization criteria.

Criterion	*n_w_* (%)	*R_C_* (MPa)	*ITS_DRY_* (MPa)	*S_m_* +10 °C (MPa)	*K_IC_* (N/mm^1,5^)
better	1.0	min	0.50	min	30.0
worse	5.0	max	0.25	max	25.0
weight	0.2	0.2	0.2	0.2	0.2
